# Genetic variation in the fat mass and obesity-associated gene (*FTO*) in association with food preferences in healthy adults

**DOI:** 10.3402/fnr.v57i0.20028

**Published:** 2013-04-12

**Authors:** Louise Brunkwall, Ulrika Ericson, Sophie Hellstrand, Bo Gullberg, Marju Orho-Melander, Emily Sonestedt

**Affiliations:** 1 Diabetes and Cardiovascular Disease – Genetic Epidemiology, Department of Clinical Sciences in Malmö, Lund University, Malmö, Sweden; 2 Nutritional Epidemiology, Department of Clinical Sciences in Malmö, Lund University, Malmö, Sweden

**Keywords:** obesity, nutrition intake, genetic, Sweden, adults, food preference

## Abstract

**Background:**

Earlier studies have indicated that the fat mass and obesity-associated gene (*FTO*) is not only associated with BMI and weight but also with appetite and dietary intake.

**Objectives:**

We investigated if the *FTO* rs9939609 associates with food preferences in healthy adults with no cancer, cardiovascular disease, or diabetes. Additionally, we challenged the question if the associations are modified by obesity status (BMI ≤25 or >25 kg/m^2^).

**Design:**

The analyses are made with 22,799 individuals from the Swedish population-based Malmö Diet and Cancer Cohort Study, who were born between 1923 and 1945. To investigate food preference, 27 food groups conducted from a modified diet history method including a 7-day registration of cooked meals and cold beverages were used in the analyses. Bonferroni correction was used to correct for multiple testing, resulting in a cut-off value for significance level of *p*<0.002.

**Results:**

We observed that the obesity susceptible A-allele carriers reported a higher consumption of biscuits and pastry but lower consumption of soft drinks (*P* for trend <0.0001 for both) as compared to TT genotype carriers. In contrast to our hypothesis, the results did not significantly differ depending on obesity status except for consumption of juice, where only the overweight individuals with A-allele had a higher consumption as compared to TT carriers (*P* for interaction=0.04).

**Conclusion:**

Our results indicate that the *FTO* A-allele may associate with certain food preference and in particular with certain energy-dense foods.

The rapidly growing number of overweight and obese people is a major public health issue worldwide and could be larger than the famine-hit. While the spreading of the obesogenic environment across the world is the main explanation to this progress the genetic susceptibility may have an important contribution to individual risk. In genome wide association studies, a number of common genetic variants associated with body mass index (BMI) and obesity have been identified, and the strongest association is found for a single nucleotide polymorphism (SNP) rs9939609 in the fat mass and obesity-associated gene (*FTO*), on chromosome 16 (1). This association has been shown in many populations (2–5), and several large studies have verified that the A-allele of rs9939609 associates with higher weight and BMI (2, 6–9).

*FTO* is expressed in the hypothalamus, and inactivation of *FTO* in mice protects against obesity (10) while over-expression leads to increased energy intake and obesity (11). In human studies, *FTO* genotype has been indicated to influence appetite regulation and food intake, especially in children and adolescents (7, 12–16). Other studies have investigated the association between *FTO* genotype and macronutrient intake (17–21), and we have previously in the Malmö Diet and Cancer Study (MDCS) observed a higher percentage of energy from protein for carriers of the A-allele (22). We also observed that high fat intake may accentuate the associating effect of the *FTO* genotype on fat mass and obesity (6, 22), but none of our previous studies have investigated the association with specific food groups. One earlier study has investigated food preferences with food groups across the *FTO* genotypes, without finding any significant differences among the studied 759 twin pairs (17).

The primary aim of this study was to investigate the association between the *FTO* genotype (rs9939609) and dietary intake from 27 food groups among men and women in the MDCS cohort. The secondary aim was to examine the associations separately in normal weight and overweight individuals, challenging the question if obesity state could modify associations between the *FTO* variant and dietary intake. The extended information about energy intake and expenditure in the present study made it possible to exclude the potential non-adequate energy reporters, which may be of particular importance as *FTO* variant has been associated with non-adequate energy reporting in a subset of the MDCS cohort (6).

## Subjects and methods

### Study participants and data collection

MDCS is a prospective cohort study that was conducted in the city of Malmö in Sweden (23). All men born between 1923 and 1945 and all women born between 1923 and 1950 in Malmö were invited via personal letters and advertisements in the local newspaper and public places during the baseline examination conducted in 1991–1996. Their age at baseline was between 44 and 74 years of age. The only exclusion criteria were mental incapacity and inadequate Swedish language skills. In total, 28,098 individuals (17,035 women and 11,063 men) provided complete dietary information and anthropometric measures, representing about 40% of the eligible individuals. All participants visited the research center on two occasions about 10 days apart. On the first visit, they received detailed instructions and information on the study, and the study material was given to them. They were all asked to fill in a menu booklet and two questionnaires: one with questions about their food habits and one with questions concerning lifestyle and socioeconomic aspects. At the first visit, nurses drew blood and anthropometrical measures were taken. On the second visit, the participants were interviewed by trained staff to complete the dietary history and to check the accuracy of the complete questionnaire. All individuals provided a written informed consent, and the ethics committee of Lund University approved the MDCS protocols.

In the present study, we have included 22,799 individuals (8,797 men and 14,002 women) who had DNA sample available and were genotyped for rs9939609, but who did not have a history of cardiovascular disease (*n*=954), cancer (*n*=1747), or diabetes (*n*=897) at the time of dietary data collection.

### Dietary data

MDCS used a modified interview-based diet history method that was specially designed for the study (24) which included: 1) a 7-day menu book for registration of cooked meals and cold beverages; 2) a 168-item questionnaire for assessment of intake frequencies and portion sizes of regularly consumed foods not covered by the menu book; and 3) a 45-min interview including more questions about the cooking methods and the product food choices. The interviewer also very carefully controlled that the answers in the questionnaire and in the 7-day menu book did not overlap. The average daily food intake (g/day) was calculated based on the questionnaire and the 7-day menu book. To convert the food intake into energy and nutrient information the MDCS database was used. The majority of the nutrient information in the MDCS database was from the PCKOST2-93 from the National Food Administration in Uppsala, Sweden. A variable was created for the season of data collection; winter (December–February), spring (March–May), summer (June–August), and fall (September–November). The processing of data was slightly altered in September 1994, and the method version variable indicates if the data was collected before or after the 1 September 1994.

### Misreporting of energy intake

By taking the reported total energy intake and energy expenditure into account, we can identify participants that may have over- or underreported their energy intake. The individually estimated physical activity level (PAL) was expressed as total energy expenditure divided with the basal metabolic rate (BMR). The total energy expenditure was calculated for each individual from the self-reported amount of physical activity at work, leisure-time physical activity, hours of household work, estimated sleeping hours, self-care, and passive time. Non-adequate energy reporters were defined as those with a ratio of reported energy intake to BMR outside 95% confidence intervals (CI) of the calculated PAL (25).

### Dietary variables

The analyzed macronutrient variables were total energy (MJ), fiber density, and percentage energy (E%) from carbohydrates, protein, fat, sucrose, saturated fatty acids (SFAs), monounsaturated fatty acids (MUFA), polyunsaturated fatty acids (PUFAs), and alcohol. The 27 food groups (g/MJ) were defined mainly depending on fat and sugar content (Appendix 1).

### Anthropometric measures

Weight was measured using a balance-beam scale. The participants wore light clothes and no shoes. Height was measured with a fixed stadiometer calibrated in centimeters. BMI was calculated from the weight in kilograms divided by the height in square meters (kg/m^2^). For categorization of BMI, we used the WHO guidelines BMI ≤25 as normal weight and >25 as overweight or obese.

### Genotyping

Genotyping of the rs9939609 was made either by matrix-assisted laser desorption ionization-time of flight mass spectrometry on the Sequenom MassARRAY platform (Sequenom, San Diego, CA, USA), or by Taqman (Applied Biosystems, Foster City, CA, USA). Genotyping was successful for 97.2% of the participants. The distribution of the rs9939609 was in Hardy–Weinberg equilibrium (*p*=0.99). The concordance rate was 0.9926.

### Statistical methods

For all the analyses we used Statistical Package of the Social Science (version 20.0; SPSS Inc., Chicago, IL, USA). The analyses were made for all individuals and for men and women separately because of a possible difference in food choices and meal patterns (26). Analyses were also performed in two BMI subgroups based on WHO guidelines, normal weight, and overweight (BMI ≤25 or >25 kg/m^2^). Two BMI groups where used to keep as much statistical power as possible, and because the median BMI in MDCS was approximately 25. We used the general linear model for continuous variables to test the differences across *FTO* genotypes. The 27 food variables were logarithmically transformed to get them normally distributed. A small value (0.001) was added before the transformation to handle zero-consumers. We also tested these variables with a non-parametric, Jonckeheere Terpsta test (27). No major differences were found for the *p*-values; therefore, the results from the parametric test were presented. In sensitivity analysis we excluded individuals classified as non-adequate energy reporters. To correct for multiple testing in the main analyses of food groups we used Bonferroni to calculate the cut-off value for significance level, 0.05/27=0.002. All analyses with dietary factors were adjusted for age, sex, season, and dietary method version. We also performed additional adjustment for BMI to exclude the possibility that the associations with food groups are secondary to association between *FTO* genotype and BMI. The interactions between the *FTO* genotype and gender and the *FTO* genotype and BMI status on intake of the food groups were assessed by introducing a multiplicative factor with the continuous variables.

## Results

### BMI, nutrient intake, and dietary reporting across the FTO genotypes

BMI and reported intake of total energy and macronutrients by the *FTO* genotype (rs9939609) are shown as background characteristics in Table 1 for all 22,799 individuals in MDCS. As reported earlier in MDCS, *FTO* associated strongly with BMI (22). We found a significant difference for protein intake (*p* for trend=0.008), sucrose (*p* for trend=0.001), and total energy intake (*p* for trend *<*0.001) across the genotypes. Carriers of the A-allele had a lower reported total energy intake and consumption of sucrose, but similar to our previous observations a significantly larger part of the energy came from protein, as compared with TT carriers (6). Significant differences were also observed for non-adequate reporting of energy across the *FTO* genotypes (*P* for trend <0.001), the highest 17% frequency of under-reporting was observed among AA-genotype carriers as compared to 15.4% among TT genotype carriers. In addition, under-reporting of energy was more common in the overweight group than in the normal weight group (19.0 vs. 11.0%, *p*<0.0001).

**Table 1:** Characteristics of the MDCS cohort according to the *FTO* rs9939609 genotype

	rs9939609				
	
Variables	TT *n*=7898	AT *n*=10982	AA *n*=3919	*P* for trend^a^	*P* for trend^b^
BMI kg/m^2^	25.4 (25.3–25.5)	25.7 (25.6–25.8)	26.0 (25.9–26.1)	<0.001	
Total energy (MJ)	9.9 (9.9–10.0)	9.8 (9.8–9.9)	9.8 (9.7–9.9)	0.001	0.046
Carbohydrates E%	45.0 (45.0–44.9)	45.0 (44.9–45.1)	44.9 (44.7–45.1)	0.379	0.398
Sucrose E%	8.6 (8.5–8.7)	8.5 (8.5–8.6)	8.4 (8.3–8.5)	0.001	0.002
Fat E%	39.3 (39.2–39.5)	39.2 (39.1–39.4)	39.3 (39.1–39.5)	0.793	0.883
SFA E%	17.0 (16.8–17.0)	16.9 (16.8–17.0)	16.9 (16.8–17.0)	0.505	0.861
MUFA E%	13.7 (16.7–16.8)	13.7 (13.6–13.7)	13.7 (13.6–13.8)	0.964	0.694
PUFA E%	6.2 (6.2–6.3)	6.2 (6.2–6.3)	6.2 (6.2–6.3)	0.848	0.913
Protein E%	15.6 (15.6–15.7)	15.7 (15.7–15.8)	15.8 (15.7–15.8)	0.008	0.006
Alcohol (g/MJ)	1.2 (1.2–1.2)	1.2 (1.2–1.2)	1.2 (1.2–1.2)	0.909	0.520

Values are for mean (95% CI).

a *P* for trend across the genotypes.

b *P* for trend across the genotypes in sensitivity analysis excluding individuals identified as non-adequate energy reporters.

All analyses are adjusted for age, sex, method, and season.

### Food preferences across *FTO* genotypes

After correcting for multiple testing, we observed significant differences by the *FTO* genotype (i.e. *p*<0.002) for reported intake of biscuits and pastry and soft drinks, where the A-allele carriers consumed more biscuits and pastry (0.07g/MJ/allele), but less soft drinks (−0.17g/MJ/allele) compared to TT-genotype carriers (Table 2). In addition, A-allele carriers reported nominally higher intakes of fruits, cereals, high fat meat, ice cream, and cheese, and a nominally lower intake of salty snacks (Table 2). The results were similar in men and women except for a significant interaction (*p*=0.003) for soft drinks; the trend was in the same direction, but stronger in women (Supplementary Table 2).

**Table 2:** The reported intake of 27 food groups in MDCS by the *FTO* rs 9939609 genotype

	rs9939609				
	
Variables	TT *n*=7898	AT *n*=10982	AA *n*=3919	*P* for trend^a^	*P* for trend^b^
Foods (g/MJ)					
Vegetables	19.2 (19.0–19.5)	19.5 (19.2–19.7)	19.4 (19.1–19.9)	0.069	0.676
Fruits	20.3 (20.0–20.6)	20.5(20.2–20.7)	20.9 (20.4–21.3)	0.017	0.256
Juice	6.3 (6.1–5.6)	6.7 (6.4–6.9)	6.1 (5.8–6.5)	0.325	0.541
Boiled potato	10.5 (10.3–10.6)	10.4 (10.3–10.5)	10.3 (10.1–10.5)	0.393	0.416
Fried potato	1.9 (1.9–2.0)	1.9 (1.9–2.0)	1.9 (1.0–2.0)	0.889	0.687
Cereals	2.0 (1.9–2.0)	2.0 (2.0–2.1)	2.0 (2.0–2.1)	0.017	0.014
Soft bread	10.8 (10.6–10.9)	10.7 (10.6–10.8)	10.7 (10.5–10.9)	0.954	0.678
Crisp bread	1.7 (1.7–1.8)	1.8 (1.7–1.8)	1.8 (1.7–1.8)	0.327	0.648
Biscuits and pastry	3.7 (3.6–3.8)	3.8 (3.7–3.8)	3.9 (3.9–4.0)	<0.001	<0.001
Rice and Pasta	1.3 (1.3–1.3)	1.3 (1.2–1.3)	1.3 (1.3–1.3)	0.659	0.534
Egg	2.5 (2.4–2.5)	2.5 (2.4–2.5)	2.5 (2.4–2.6)	0.548	0.817
Meat low fat	7.1 (7.0–7.2)	7.1 (7.0–7.2)	7.1 (7.0–7.3)	0.395	0.818
Meat high fat	4.3 (4.3–4.4)	4.4 (4.4–4.5)	4.4 (4.3–4.5)	0.006	0.024
Fish low fat	2.9 (2.8–3.0)	2.9 (2.9–3.0)	3.0 (2.9–3.1)	0.207	0.628
Fish high fat	1.9 (1.9–2.0)	1.9 (1.9–2.0)	1.9 (1.8–2.0)	0.842	0.957
Milk low fat	23.2 (22.6–23.7)	23.8 (23.3–24.3)	23.6 (22.8–24.4)	0.054	0.048
Milk high fat	14.8 (14.4–15.2)	14.5 (14.1–14.8)	14.1 (13.5–14.6)	0.262	0.664
Cream	1.5 (1.5–1.5)	1.5 (1.5–1.6)	1.6 (1.6–1.6)	0.057	0.150
Ice cream	20.3 (20.0–20.6)	20.5 (20.2–20.8)	20.9 (20.4–21.3)	0.018	0.259
Margarine high fat	19.2 (19.0–19.5)	19.5 (19.3–19.7)	19.5 (19.1–19.9)	0.068	0.663
Margarine low fat	1.8 (1.7–1.8)	1.8 (1.8–1.9)	1.8 (1.7–1.9)	0.056	0.468
Cheese	4.3 (4.3–4.4)	4.4 (4.4–4.5)	4.4 (4.3–4.5)	0.006	0.024
Soft drinks	8.5 (8.2–8.8)	8.2 (8.0–8.5)	7.7 (7.2–8.1)	<0.001	<0.001
Soft drinks no energy	1.1 (0.98–1.3)	1.3 (1.1–1.4)	1.2 (0.96–1.4)	0.428	0.868
Sugars and Sweets	3.4 (3.4–3.5)	3.3 (3.3–3.4)	3.3 (3.2–3.4)	0.232	0.479
Chocolate	0.8 (0.8–0.8)	0.8 (0.8–0.8)	0.8 (0.8–0.8)	0.634	0.641
Salty snacks	0.02 (0.02–0.02)	0.02 (0.02–0.02)	0.01 (0.01–0.02)	0.014	0.126

Values are for mean (95% CI).

a *P* for trend across the genotypes.

b *P* for trend across the genotypes in sensitivity analysis excluding individuals identified as non-adequate energy reporters.

All analyses are adjusted for age, sex, method, and season.

### Food preferences across the *FTO* genotypes in strata of BMI

When we stratified for BMI, the trends across the *FTO* genotypes were similar except for the consumption of juice (*p*-interaction=0.04), which indicated a nominally significant increased intake for the A-allele carriers among overweight individuals but not among the normal weight individuals (Table 3).

**Table 3:** The reported intake of 27 food groups by the *FTO* rs9939609 genotype in normal weight and overweight participants of MDCS

	rs9939609									

		BMI ≤25					BMI >25				
		
Variables	TT *n*=3995	AT *n*=5218	AA *n*=1721	*P* a	*P* b	TT *n*=3892	TA *n*=5748	AA *n*=2197	*P* for trend^a^	*P* for trend^b^
Foods (g/MJ)										
Vegetables	18.7	19.0	19.1	0.108	0.722	19.8	20.0	19.8	0.665	0.982
Fruits	19.0	19.2	20.1	0.028	0.206	21.5	21.6	21.4	0.561	0.998
Juice	6.5	6.7	5.9	0.458	0.158	6.1	6.6	6.3	0.029	0.024
Boiled potato	10.3	10.1	9.9	0.232	0.304	10.6	10.6	10.7	0.928	0.876
Fried potato	1.9	1.9	1.8	0.175	0.205	1.9	1.9	2.0	0.363	0.644
Cereals	2.1	2.1	2.1	0.155	0.476	1.9	2.0	2.0	0.016	0.002
Soft bread	11.1	11.1	11.1	0.920	0.400	10.5	10.4	10.4	0.808	0.279
Crisp bread	1.7	1.7	1.7	0.594	0.541	1.8	1.8	1.9	0.095	0.250
Biscuits and pastry	3.7	3.8	4.0	0.005	0.002	3.7	3.7	3.9	0.004	0.031
Rice and Pasta	1.3	1.3	1.2	0.164	0.273	1.3	1.3	1.3	0.398	0.696
Egg	2.4	2.4	2.3	0.287	0.878	2.5	2.6	2.6	0.488	0.805
Meat low fat	6.8	6.8	6.8	0.974	0.386	7.5	7.4	7.3	0.330	0.221
Meat high fat	4.4	4.5	4.5	0.078	0.370	4.2	4.3	4.3	0.036	0.028
Fish low fat	2.8	2.8	2.9	0.504	0.835	3.0	3.0	3.1	0.287	0.391
Fish high fat	1.9	1.8	1.9	0.720	0.669	2.0	2.0	1.9	0.951	0.595
Milk low fat	21.0	21.0	21.1	0.353	0.383	25.4	26.3	25.5	0.235	0.151
Milk high fat	15.5	15.3	14.5	0.900	0.603	14.1	13.7	13.7	0.300	0.359
Cream	1.5	1.6	1.6	0.185	0.359	1.5	1.5	1.5	0.083	0.197
Ice cream	19.0	19.2	20.1	0.026	0.201	21.4	21.6	21.4	0.587	0.975
Margarine hi	18.7	19.0	19.1	0.108	0.375	19.8	20.0	19.8	0.660	0.978
Margarine low	1.7	1.8	1.8	0.220	0.573	1.8	1.9	1.8	0.347	0.896
Cheese	4.4	4.5	4.5	0.078	0.370	4.3	4.3	4.3	0.002	0.028
Soft drinks	7.8	7.4	7.2	<0.001	0.001	9.1	8.9	8.0	0.019	0.019
Soft drink no e	0.82	0.97	0.73	0.673	0.351	1.5	1.5	1.5	0.486	0.938
Sugars and sweets	3.6	3.5	3.5	0.593	0.673	3.3	3.2	3.1	0.630	0.328
Chocolate	0.80	0.80	0.85	0.440	0.572	0.79	0.79	0.78	0.147	0.248
Salty Snacks	0.02	0.02	0.02	0.269	0.642	0.02	0.02	0.01	0.017	0.642

Values are for mean (95% CI).

a *P* for trend across the genotypes.

b *P* for trend across the genotypes in sensitivity analysis excluding individuals identified as non-adequate energy reporters.

All analyses are adjusted for age, sex, method, and season.

In the sensitivity analysis only including individuals classified as adequate energy reporters, our results for food intakes across *FTO* genotypes remained virtually unchanged, except for intakes of fruits and salty snacks, which did not remain nominally significant. An additional adjustment for BMI did not change any of the reported results.

## Discussion

The *FTO* genotype is believed to influence appetite through actions in the hypothalamus where it is expressed in parts that control hunger and satiety (7, 8, 15, 16). Our study suggests that the A-allele may not only associate with appetite in general, but also with preference for specific food groups. Individuals carrying the A-allele were observed to consume significantly more biscuits and pastry and less soft drinks compared with TT carriers. In addition, we found nominal differences in reported consumption of fruits, cereals, high fat meat, ice cream, cheese, and salty snacks where the A-allele carriers reported a higher consumption of these food groups except for salty snacks where they reported a lower consumption. We did not observe any major differences in results between men and women or between normal weight and overweight individuals except that juice intake was nominally higher among overweight A-allele carriers.

Several studies, including one of our previous, have investigated macronutrient intake across *FTO* genotypes where we, in line with this study, found AA carriers to have a significantly increased protein intake (*p*=0.008). However, this association could not easily be explained by differences in intake of protein-rich food groups. Only one previous study has investigated food prevalence with food groups, and that study investigated intake levels of 20 food groups’ in 756 adult twin pairs without finding any significant associations with *FTO* genotype. However interestingly, in line with our study, that study reported a tendency towards a difference for soft drink consumption across the *FTO* genotypes where the AA-genotype carriers consumed less soft drinks than the TT-genotype carriers (*P* for trend=0.07) (17). A lower intake of soft drinks in our study may at least partially explain the observed lower sucrose intake among A-allele carriers (*P* for trend=0.001) although soft drinks were not as highly consumed at the time of the baseline examinations as they are today. Other studies have detected an association between the *FTO* genotype and BMI among individuals consuming a diet rich in fat and especially in saturated fat (12, 13, 18, 20). Some of the food groups that indicated differences across the *FTO* genotypes in our study also contain a high amount of carbohydrates, but considering our findings of a lower intake of sucrose among the A-allele carriers, it may be considered more likely that it is the increased intake of energy-dense products that may contribute to the increased obesity risk in the risk allele carriers.

In the present study, we observed an increased intake of foods usually consumed in addition to the main meals during a day by the A-allele carriers, i.e. biscuits and pastry. In addition, most of the food groups that indicated nominally increased consumption among A-allele carriers were foods that might be consumed in addition to main meals like fruits, ice cream, cereals, and cheese. In line with these results, a study that investigated food patterns across the *FTO* genotypes found the A-allele carriers to consume a higher number of meals per day and to eat more servings of energy-dense foods (28). Consistent with our results a study of 4–5 year old children who were served unrestricted amount of biscuits for 10 min after a main meal reported that children carrying the A-allele ate significantly more, which led to the conclusion that the A-allele carriers are potentially less responsive to internal signals of satiety compared to TT-genotype carriers (14). In another study, where 4–10-year-old children were served juice and 56 g muffins with different energy contents after a main meal, the A-allele carrying children demonstrated a higher energy intake and a preference for energy-dense foods (7). Further, a study focusing on *FTO* genotype and its putative association with cognitive effects reported that children carrying the A-allele suffered from loss of control over eating to a higher extent than the TT-genotype carriers (29). It is obvious that increased preference for energy-dense foods, increased number of meals per day, a less sensitive satiety signaling, and a loss of control over eating all contribute to an increased weight and, in a longer perspective, lead to overweight and obesity and may explain the association between *FTO* and obesity in our population.

Our observations of an increased intake of biscuits and pastry could be specific for this population depending on the age of the individuals and the geographic location of the study. In other populations it could be another energy-dense food group. We would also like to point out that even if the association is significant, the increased amount of biscuits and pastry that the A-allele carrying individuals consume is very small (0.07g/MJ/allele, corresponding to approximately 1.4 g higher intake in TT compared with AA carriers). So, if this has an impact on the obesity risk for the AA carrying individuals it is extremely small. However, if our findings can be replicated in other populations and used together with findings from other obesity genes this could play a part in future obesity prevention and care.

We have a high relative validity of the diet data and a large number of subjects compared with other studies made on this topic so far, which gives us high power to detect even weaker associations. Still our study suffers from some limitations that need to be discussed. Misreporting of energy is a major concern in nutritional epidemiology and is also a limitation of our study. It is well known that obese individuals tend to under-report their energy intake to a higher extent than lean individuals (30). In addition to this we observed that under-reporting was more common among the A-allele carriers than among the TT carriers (6), which may also explain the lower reported total energy intake among the AA-genotype carriers. However, it needs to be kept in mind that calculated under-reporting of energy can also be a consequence of over-reporting of physical activity or a consequence of an individual having a lower basal metabolic rate (BMR/kg). In fact, the *FTO* A-allele was recently reported to associate with lower BMR/kg (31) while another study using measured BMR could not find such associations (32). The physical activity in the present study was based on self-reported answers to a questionnaire that may be hard to answer even if it has been validated (33). However, due to the extended information on both dietary intake and physical activity we are able to exclude the potential inadequate reporters in the sensitivity analysis. Our results did not virtually differ after exclusion of potential miss-reporters except for consumption of fruits, which did not remain nominally higher among A-allele carriers after the exclusion. In this context, it is interesting that an earlier report from MDCS observed that high consumption of fruits was associated with under-reporting of energy (34).

The individuals in this study were born in between 1923 and 1950 when the possibilities for selection and accessibility of food were far from what it is today. These individuals can be expected to be characterized by meal patterns different from younger individuals today. In addition, our study population did not grow up in the obesogenic environment we have today, which might contribute with an attenuated genetic susceptibility as compared to studies made on younger subjects (35). Despite this, we were able to detect differences in consumption of some of the food groups across the *FTO* genotypes.

In conclusion, in this large cohort of 22,799 individuals we observed that the *FTO* rs9939609 associates weakly but significantly with higher intake levels of biscuits and pastry and lower levels of soft drinks. This may indicate that *FTO* associates with certain food preference and in particular with energy-dense foods.
